# Characterization and identification of lysine glutarylation based on intrinsic interdependence between positions in the substrate sites

**DOI:** 10.1186/s12859-018-2394-9

**Published:** 2019-02-04

**Authors:** Kai-Yao Huang, Hui-Ju Kao, Justin Bo-Kai Hsu, Shun-Long Weng, Tzong-Yi Lee

**Affiliations:** 1 School of Science and Engineering, The Chinese University of Hong Kong, Shenzhen, 518172 China; 2 Warshel Institute for Computational Biology, The Chinese University of Hong Kong, Shenzhen, 518172 China; 3 Department of Computer Science and Engineering, Yuan Ze University, Taoyuan city, 320 Taiwan; 4 Department of Medical Research, Taipei Medical University Hospital, Taipei city, 110 Taiwan; 5 Department of Medicine, Mackay Medical College, New Taipei City, 252 Taiwan; 6 Mackay Medicine, Nursing and Management College, Taipei, 112 Taiwan; 7 Department of Obstetrics and Gynecology, Hsinchu Mackay Memorial Hospital, Hsin-Chu, 300 Taiwan

**Keywords:** Protein glutarylation, Intrinsic interdependence, Maximal dependence decomposition

## Abstract

**Background:**

Glutarylation, the addition of a glutaryl group (five carbons) to a lysine residue of a protein molecule, is an important post-translational modification and plays a regulatory role in a variety of physiological and biological processes. As the number of experimentally identified glutarylated peptides increases, it becomes imperative to investigate substrate motifs to enhance the study of protein glutarylation. We carried out a bioinformatics investigation of glutarylation sites based on amino acid composition using a public database containing information on 430 non-homologous glutarylation sites.

**Results:**

The TwoSampleLogo analysis indicates that positively charged and polar amino acids surrounding glutarylated sites may be associated with the specificity in substrate site of protein glutarylation. Additionally, the chi-squared test was utilized to explore the intrinsic interdependence between two positions around glutarylation sites. Further, maximal dependence decomposition (MDD), which consists of partitioning a large-scale dataset into subgroups with statistically significant amino acid conservation, was used to capture motif signatures of glutarylation sites. We considered single features, such as amino acid composition (AAC), amino acid pair composition (AAPC), and composition of k-spaced amino acid pairs (CKSAAP), as well as the effectiveness of incorporating MDD-identified substrate motifs into an integrated prediction model. Evaluation by five-fold cross-validation showed that AAC was most effective in discriminating between glutarylation and non-glutarylation sites, according to support vector machine (SVM).

**Conclusions:**

The SVM model integrating MDD-identified substrate motifs performed well, with a sensitivity of 0.677, a specificity of 0.619, an accuracy of 0.638, and a Matthews Correlation Coefficient (MCC) value of 0.28. Using an independent testing dataset (46 glutarylated and 92 non-glutarylated sites) obtained from the literature, we demonstrated that the integrated SVM model could improve the predictive performance effectively, yielding a balanced sensitivity and specificity of 0.652 and 0.739, respectively. This integrated SVM model has been implemented as a web-based system (MDDGlutar), which is now freely available at http://csb.cse.yzu.edu.tw/MDDGlutar/.

**Electronic supplementary material:**

The online version of this article (10.1186/s12859-018-2394-9) contains supplementary material, which is available to authorized users.

## Background

Protein post-translational modifications (PTM) consist of chemical modifications that play an important role in many cellular processes in biology, such as regulating the activity, localization, and protein interactions. Some PTMs occur at amino acid side chains of a protein molecule, generally by covalent enzymatic modification. Most PTMs are enzymatically controlled and regulated. Phosphorylation is the most well-known example of a PTM, which is the attachment of a phosphoryl group to a serine (S), threonine (T), or tyrosine (Y) residue of a protein via protein kinases [1]. Crucial protein PTM occurring at the ε-amino groups of specific lysine residues (K) have proven to be hallmarks of active chromatin, and major regulators of gene expression, protein-protein interactions, and protein processing and degradation. These include 2-hydroxyisobutyrylation [2], acetylation [3], butyrylation [4], crotonylation [5], malonylation [6], propionylation [7] and succinylation [8]. In addition, malonylation, succinylation, and glutarylation, which are collectively referred to as lysine acylation, are highly dynamic PTMs and appear conserved across evolutionarily related species [3].

Lysine glutarylation, which is the addition of a glutaryl group to a lysine residue of a protein molecule, is an important posttranslational modification. It plays a crucial role in mitochondrial functions and metabolic processes both in eukaryotic and prokaryotic cells, such as amino acid metabolism, fatty acid metabolism and cellular respiration [6]. Previous studies have indicated that the activity of carbamoyl phosphate synthase 1 was inhibited through protein glutarylation [9]. However, lysine acyltransferases (KATs) can bind specificity to its substrates in malonylation and succinylation but lacking evidence for glutarylation. Because of the similarities in biological psychology, we surmised the mechanism of glutarylation in much the same way that can be enzymatically catalyzed by KATs and removed by lysine deacylases (KDACs) as acylation. Moreover, owing to the labile nature and low abundance of in vivo glutarylation sites, further research is needed to clarify the characteristics of glutarylation and its mechanisms. Therefore, there is an urgent requirement in bioinformatics for a practical approach to investigate the potential substrate motifs of protein glutarylation sites to be designed.

In this study, we used a similar concept as that previously developed to predict protein functional sites using in silico characterization of substrate specificity [10–14]. We selected sequence-based features to discriminate between glutarylation sites and non-glutarylation sites, such as amino acid composition (AAC), amino acid pair composition (AAPC), and composition of k-spaced amino acid pairs (CKSAAP). Additionally, we applied a chi-square test, as part of maximal dependence decomposition (MDD), to measure the interdependence between positions in the substrate sites. Further, MDD can partition large-scale PTM sites data into subgroups according to the most significant dependencies between amino acid compositions surrounding the substrate sites. We explored promising consensus motifs for glutarylation sites by applying MDD [15]. Subsequently, for each subgroup containing one of the MDD-identified substrate motifs, we built a predictive model using support vector machine (SVM). Furthermore, to assess the effectiveness of the proposed models by five-fold cross-validation, we created an independent test dataset by extracting experimental data from the literature, which was completely blind to the training dataset. To facilitate the study of protein glutarylation, we are motivated to design a public system, named MDDGlutar (http://csb.cse.yzu.edu.tw/MDDGlutar/), for the identification of glutarylation sites and their corresponding motifs using experimentally verified glutarylation sites curated from research articles.

## Methods

### Data collection and preprocessing

Protein Lysine Modifications Database (PLMD) [16] is a manually curated database of experimentally verified glutarylation sites, which contains 715 glutarylation sites of 211 proteins. The purpose of this study was to investigate potential substrate motifs based on the amino acids surrounding glutarylated lysine residues. For this reason, we extracted sequence fragments centered around experimentally verified glutarylation sites with a window length of 2n + 1, such that the fragment included n upstream and n downstream flanking amino acids. These sequence fragments of length 2n + 1 amino acids centered at the glutarylated lysine residue were regarded as the positive dataset. Alternatively, if the lysine residue has not been annotated as a glutarylation site, the fragments were regarded as the negative dataset. The determination of an appropriate window size for model construction is difficult without the well-defined information of motif signatures. Hence, we adopted different window lengths ranging from 11 to 25 for the preparation of training datasets. The performance comparison among predictive models using different window lengths was performed on the basis of SVM classifier with AAC features. As shown in Additional file 1, the cross-validation results displayed that the model trained using 21-mer window length could provide best performance than that using other window lengths.

The CD-HIT software [17] is a useful tool for clustering protein sequences based on a specified value of sequence identity. It was used to remove homologous sequence fragments from the positive and negative datasets, preventing overestimation of the predictive performance. Because of the incomplete information available concerning the experimentally validated glutarylation sites, analysis of sequence fragments could sometimes lead to a negative sequence appearing identical to a positive sequence, potentially causing false positive or false negative predictions. Therefore, CD-HIT was applied a second time by running cd-hit-2d across the positive and negative training datasets with 100% sequence identity. If a sequence in the negative set was the same as a sequence in the positive set, only the sequence in the positive set was reserved, and the sequence in the negative set was discarded. After filtering out homologous fragments with 40% sequence identity, as shown in Table 1, the non-homologous dataset consisted of 476 positive sequences and 1918 negative sequences. The non-homologous dataset was divided into two parts, training dataset and independent dataset. The training dataset included 430 positive sequences, and a random selection of approximately 1:2 of the 860 negative sequences (approximately the ratio between the number of positive and negative sequences). The remaining sites were used as the testing dataset. Based on the binary classification, the positive (glutarylation sites) and negative (non-glutarylation sites) datasets were used to build a predictive model.

**Table 1:** Data statistics of training and testing datasets after the removal of homologous sequences using CD-HIT program

Sequence identity cut-off	Number of glutarylation sites	Number of non-glutarylation sites
Raw Data	715	4145
90%	667	3675
80%	631	3317
70%	597	3037
60%	556	2767
50%	534	2539
40%	476	1918
Training data	430	860
Independent testing data	46	92

However, since the parameters of the predictive model were optimized, its predictive performance might be overestimated because of over-fitting the training dataset. In order to evaluate the actual performance of the proposed models, we generated an independent dataset, blind to the training datasets. The independent testing dataset was generated by extracting glutarylated peptides excluding those represented in training dataset. Similar to the training dataset, based on a window size of 21 (*n* = 10), the independent testing dataset contained 46 positive and 92 negative sequences (Table 1).

### Investigation and encoding of sequence-based features

The research in this study focused on the analysis of sequence-based features including amino acid composition (AAC), amino acid pair composition (AAPC), and composition of k-spaced amino acid pairs (CKSAAP). To construct an SVM prediction model, fragment sequences must be transformed into numeric vectors based on various features. The training dataset contains *k* vectors {***x***_***i***_, ***i*** **= 1**, **2**…, ***k***}, which represent the k sequence fragments of length corresponding to the specified window length. To classify sites as glutarylation and non-glutarylation sites, a label was applied to each data to mark the class of its corresponding protein.

Amino acid composition (AAC) is a widely-used sequence feature for calculating the occurring frequency of twenty amino acids within a given sequence fragment. There are 21 types of amino acids that need to be considered for feature encoding. The vector ***x***_***i***_ represented the 20 native amino acids and 1 rare amino acid. Some rare amino acids and non-existing “X” residues were used to represent less than 21-mer fragment sequences at an N- or C-terminus [18]. Given a sequence fragment *k*, ***f***_***k***_(*n*) represents the number of occurrences of native amino acids, where *n* stands for one of the 20 types of native amino acids. Hence, the composition for each of the twenty amino acids ***P***_***k***_(*n*) is computed as follows [19]:

$$ {P}_k(n)=\frac{f_k(n)}{\sum_{n=1}^{20}{f}_k(n)}n=1,2,\dots, 20. $$

The AAC vector of a sequence fragment ***x***_***k***_ is then define as

$$ {x}_k=\left[{P}_k(1),{P}_k(2),\dots, {P}_k(20)\right]. $$

To encode the composition of each of the twenty amino acids around the glutarylation sites, the 21-dimensional vector ***x***_***k***_ included 21 elements specifying the frequencies of 21 amino acids normalized by the total number of amino acids in a fragmented sequence. The composition of amino acid pairs (AAPC) [20], transforms a sequence fragment into a 441-dimensional vector, which includes 441 elements specifying the numbers of occurrences of 441 amino acid pairs divided by the total number of amino acid pairs in a fragmented sequence [21]. CKSAAP [22] is a widely used sequence encoding method that has been applied with great success to many PTM prediction problems, such as O-glycosylation [23], palmitoylation [24], ubiqutination [8], phosphorylation [25], pupylation [26], methylation [27], N-formylation [28] and crotonylation [29]. In this study, we also employed CKSAAP to classify lysine residues into glutarylation and non-glutarylation sites. For example, a pair between glycine (G) and alanine (A), separated by one (*k* = 1) amino acid of any type, is represented as GxA. To represent a sequence fragment, for each *k* (*k* = 1, 2 and 3), the 441-dimensional feature vector ***x***_***k***_ needs to be computed, where each component is the frequency of the corresponding k-spaced amino acid pair appearing in this sequence fragment, respectively.

### Capturing the intrinsic interdependence between positions

Following previously described methods [30], a dependency graph model was developed to fully capture the intrinsic interdependence between base positions in a DNA splice site. Hence, we used a chi-square test, as employed in maximal dependence decomposition (MDD) [31], to measure the interdependence between positions in the substrate sites. To perform the dependence testing on a pair of amino acids at the *i*-th and *j*-th positions of a glutarylated site, we built a 21 × 21 matrix by counting, from a sample of fragment sequences, the observed number ***Y***_***mn***_ of fragment sequences where the *i*-th amino acid ***X***_***i***_ was *m* and the *j*-th amino acid ***X***_***j***_ was *n.* The test statistic used can be described as follows:

$$ {\upchi}^2\left({X}_i,{X}_j\right)=\sum \limits_{m=1}^{21}\sum \limits_{n=1}^{21}\frac{{\left({Y}_{mn}-{E}_{mn}\right)}^2}{E_{mn}} $$

where

$$ {E}_{mn}=\frac{Y_{mc}{Y}_{rn}}{Y} $$

***Y***_***mc***_ and ***Y***_***rn***_ are row sums and column sums of the matrix, respectively. ***E***_***mn***_ is the expected number of fragment sequences, from a sample of fragment sequences, in which the *i*-th amino acid ***X***_***i***_ is *m* and the *j*-th amino acid ***X***_***j***_ is *n*. We have selected a window size of 21-mer fragment sequences in the training data to capture the intrinsic interdependence between positions. The amino acids upstream to the glutarylated lysine (***P***_**0**_) were marked as position ***P***_***−*10**_ to ***P***_***−*1**_, whereas those downstream were marked as positions ***P***_***+*1**_ to ***P***_***+*10**_. After constructing, from a glutarylated site, the matrix for each pair (***P***_***i***_,***P***_***j***_) of amino acids at distinct positions of the glutarylated site, we measured the independence for ***P***_***i***_ and ***P***_***j***_ with the chi-square test **χ**^**2**^(***P***_***i***_, ***P***_***j***_). As stated previously, the proposed method can fully capture the intrinsic interdependence between two positions surrounding glutarylation sites.

### Detection of motif signatures by maximal dependence decomposition

Three types of lysine modifications have been recently described which are collectively referred to as lysine acylation: malonylation, succinylation, and glutarylation. A previous study has reported sirtuin 5 (SIRT5) as a lysine deacylase (KDAC) that has potent demalonylase, desuccinylase and deglutarylase activities, both in vitro and in vivo [9, 32, 33]. In contrast, it is worth noting that protein malonylation and succinylation can be enzymatically catalyzed by lysine acyltransferases (KATs). Despite the lack of direct evidence that KAT enzymes bind specificity to its substrates and results in glutarylation. Because of the similarities in biological psychology, we surmised the mechanism of glutarylation in much the same way that a substrate can be glutarylated by one or more KATs.

The increasing number of experimentally identified glutarylation peptides warrants further investigation of substrate motifs to facilitate the study of protein. However, no tool was available so far to predict glutarylation sites, and analysis of their respective substrate motifs remains limited. Thus, the aim of this study was to explore motif signatures of protein glutarylation based on the amino acids surrounding substrate sites. Maximal dependence decomposition (MDD) [31] was utilized to cluster all fragment sequences into subgroups in order to detect those motifs that were statistically conserved among largescale sequence data. The clustering method was performed using MDDLogo [15], which has been demonstrated to increase the effectiveness of PTM sites identification by dividing a group of protein sequences into smaller subgroups before performing the computational identification of the PTM sites [21, 34–44]. In this investigation, a chi-square test **χ**^**2**^(***A***_***i***_, ***A***_***j***_) was used to iteratively evaluate the interdependence between two positions, ***A***_***i***_ and ***A***_***j***_, which are flanking the substrate site, based on the occurrence of amino acids. Amino acids, 20 in total, were categorized into 5 groups, based on their biochemical properties: polar, acidic, basic, hydrophobic, and aromatic. A contingency table describes the frequency of the presence of each of the twenty amino acids in positions ***A***_***i***_ and ***A***_***j***_. The chi-square test was defined as:

$$ {\upchi}^2\left({A}_i,{A}_j\right)=\sum \limits_{m=1}^5\sum \limits_{n=1}^5\frac{{\left({X}_{mn}-{E}_{mn}\right)}^2}{E_{mn}} $$

where *X*_*mn*_ is the number of target sequences having amino acids of group *m* in position *A*_*i*_ and having amino acids of group *n* in position *A*_*j*_, for each pair (*A*_*i*_, *A*_*j*_) and *i* ≠ *j*. *E*_*mn*_ was determined as $\frac{X_{nR}-{X}_{Cn}}{X}$, where *X*_*mR*_ = *X*_*m*1_ + … + *X*_*m*5_, *X*_*Cn*_ = *X*_1*n*_ + … + *X*_5*n*_ and *X* stands for the total number of target sequences. If there is a significant dependence (determined as a *X*^2^ value higher than 34.3, proportional to a cutoff level of *P* = 0.01 with 16 degrees of freedom) between two positions, it followed the description of Burge and Karlin [31]. After the recursive chi-square testing, MDD algorithm can divide a group of target sequences into subsets that capture the most significant dependencies of positions on each other. When executing MDDLogo, a parameter, i.e., the maximum cluster size, should be set. If the size of a subgroup is less than the specified value of maximum cluster size, the subgroup will not be divided any further. The clustering process of MDD shall be terminated when all the subgroup sizes are less than the specified value of maximum cluster size [15].

### Construction of predictive model

**Fig. 1:**
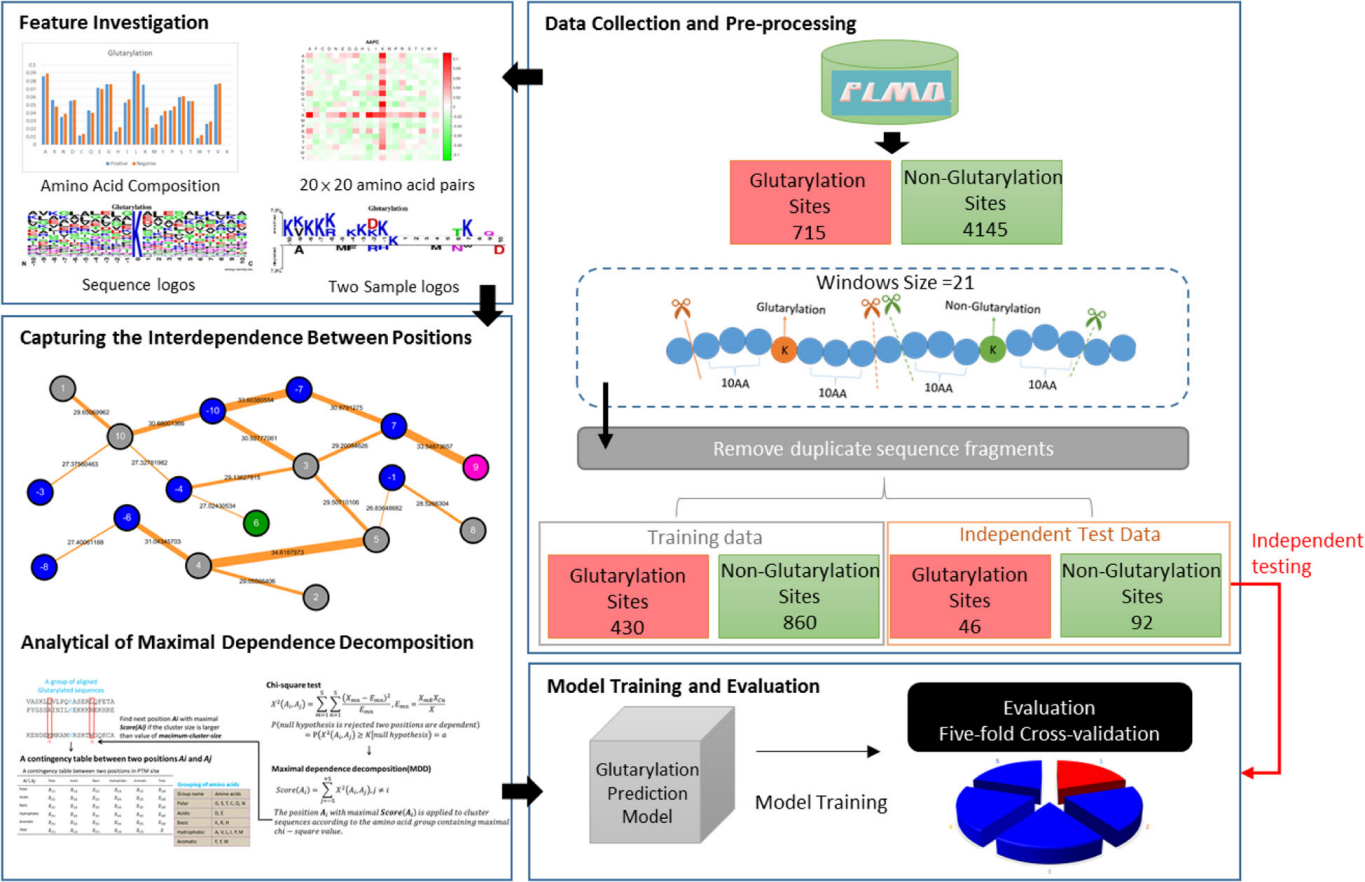
Flowchart of performing protein glutarylation site prediction in this work. It mainly consists of data collection and preprocessing, feature investigation, determination of intrinsic interdependence between positions of substrate sites, detection of motif signatures, model training and evaluation, and independent testing

A support vector machine (SVM) [45] is a discriminative classifier for pattern recognition and data classification. As shown in Fig. 1, we employed a public SVM library (LIBSVM) [46] to implement the predictive model for distinguishing glutarylation sites from non-glutarylation sites. Based on this binary classification of samples as positive or negative, a kernel function transformed the input samples into a higher dimensional space. Subsequently, a hyperplane is determined for discriminating between the two classes with maximal margin and minimal error by a separating hyperplane. As described in a number of previous works [21, 47–52], the radial basis function (RBF): defined as (***S***_***i***_, ***S***_***j***_) **=  exp** (**−γ**‖***S***_***i***_ **−** ***S***_***j***_‖^**2**^), is a reasonably best choice for SVM classifier learning. The RBF kernel is determined by a gamma (**γ**) parameter, while the cost © parameter controls the hyper-plane softness. The two supporting parameters could be optimized by a Python program (grid.py) of LIBSVM to improve the predictive accuracy. In this study, LIBSVM was applied to build a predictive model for each feature, and the best feature was selected as the training feature to construct a predictive model for each MDD-clustered subgroup.

### Evaluation of predictive performance

We evaluated the predictive performance of the proposed models trained with various features using five-fold cross-validation. First, the training data were randomly divided into five subgroups of approximately equal size, one was used as validation data and the remaining four subgroups were used as training data. The five validation results were then combined to generate a single estimation. Cross-validation evaluation improves the reliability of evaluation, because it considers all the original data, both from the training and testing data sets, and tests each subset only once [47]. To gauge the effective predictive performance of the training model, the following measures were used: sensitivity (Sn), specificity (Sp), accuracy (Acc) and Matthews Correlation Coefficient (MCC):

$$ {\displaystyle \begin{array}{c} Sn=\frac{TP}{TP+ FN}\\ {} Sp=\frac{TN}{TN+ FP}\\ {} Acc=\frac{TP+ TN}{TP+ FN+ TN+ FP}\\ {} MCC=\frac{\left( TP\times TN\right)\hbox{-} \left( FN\times FP\right)}{\sqrt{\left( TP+ FN\right)\times \left( TN+ FP\right)\times \left( TP+ FP\right)\times \left( TN+ FN\right)}}\end{array}} $$

where TP, TN, FP, and FN represent the numbers of true positives, true negatives, false positives and false negatives, respectively. To evaluate how well the model distinguished between glutarylation sites and non-glutarylation sites, five-fold cross-validation was used to assess the predictive performance of the models. To compare with other approaches, we have utilized five-fold cross validation to evaluate the performance of the proposed method. Meanwhile, we also test the stability of predictive performance by using other *k* values (ranging from 6 to 10) for *k*-fold cross validation. The results presented in Additional file 2 indicated that the SVM model trained with AAC feature could provide a stable performance on different *k* values of *k*-fold cross validation. The predictive model with the best performance in the cross-validation evaluation was considered as a final model. Finally, an independent test was carried out on this final model.

## Results

### Composition of amino acids around glutarylation sites

**Fig. 2:**
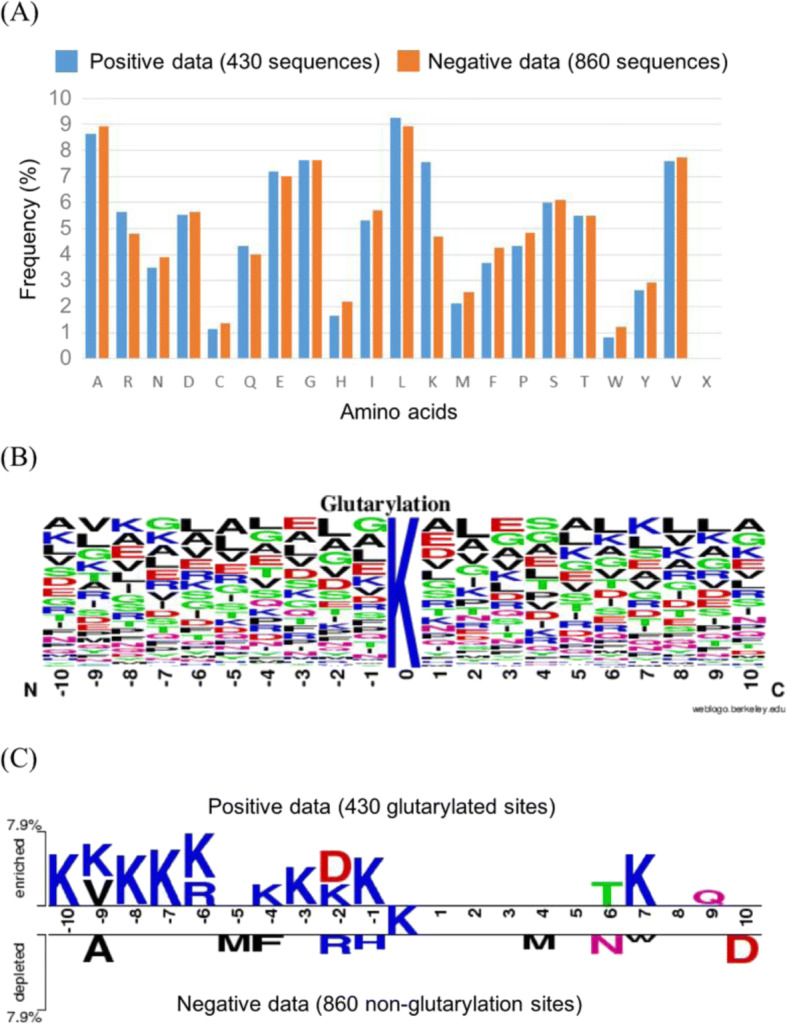
Composition of amino acids around glutarylation sites. **a** Comparison of AAC between 430 positive and 860 negative sequences. **b** Position-specific AAC of 430 glutarylated sequences. **c** Comparison of position-specific AAC between glutarylated and non-glutarylated sequences based on TwoSampleLogo analysis

To explore potential consensus motifs, the frequency of occurrence around glutarylation sites of each of the 20 amino acids was investigated based on 430 fragment sequences using a 21-mer window length. Fig 2a indicates that, at glutarylation sites, lysine (K) and arginine ® residues occur more frequently, while asparagine (N), histidine (H), methionine (M), phenylalanine (F), and proline (P) residues occur less frequently. Additionally, WebLogo [53] was utilized to compute the position-specific amino acid composition for glutarylation sites (Fig. 2b). However, it is difficult to compare the amino acid composition between glutarylation and non-glutarylation sites at a specific position. Thus, the TwoSampleLogo tool [54] was employed to detect differences in position-specific symbol compositions between the glutarylated and non-glutarylated instances. Lysine was placed in the middle of the fragment sequences, and positions of the flanking amino acids ranged from − 10 to + 10. Comparison of the 430 glutarylated sites and 860 non-glutarylated sites in Fig. 2c indicates that positively charged amino acids such as lysine (K) residues had the highest ratios at position + 7, upstream on the peptide compared to the glutarylation site (with *P* < 0.01). It also shows that polar amino acids such as threonine (T) and glutamine (Q) are slightly more abundant than expected at positions + 6 and + 9. Position − 2 was a special case, exhibiting the highest proportion of acidic residues; namely aspartate (D). This analysis shows that, in a sequence, the distance between amino acid with different properties plays a vital role in distinguishing between glutarylated and non-glutarylated sequences.

### Performance evaluation of the trained models

To determine sequence-based features to discriminate between glutarylation sites and non-glutarylation sites, SVM models were built using various sequence-based features, including AAC, AAPC and CKSAAP. Each predictive model was evaluated using five-fold cross-validation based on four measures: sensitivity (Sn), specificity (Sp), accuracy (Acc), and Matthews correlation coefficient (MCC). As shown in Table 2, the SVM model trained with AAC had the highest MCC, 0.22, and relatively high sensitivity, specificity, and accuracy, with values of 0.62, 0.61, and 0.62, respectively. The SVM model trained using AAPC with a 441-dimensional vector did not perform well, with a sensitivity of 0.61, specificity of 0.48, accuracy of 0.53, and MCC value of 0.09. On the other hand, the composition of 3-spaced amino acid pairs (CKSAAP) was found to be the worst feature for predicting glutarylation sites, with a sensitivity of 0.66, specificity of 0.41, accuracy of 0.49, and MCC of 0.07.

**Table 2:** Five-fold cross validation results on SVM models trained with various features

Training features	Sensitivity	Specificity	Accuracy	MCC
Amino Acid Composition (AAC)	62.0%	61.3%	61.6%	0.22
Amino Acid Pair Composition (AAPC)	61.3%	48.1%	52.5%	0.09
CKSAAPª, K = 1	62.0%	51.7%	55.1%	0.13
CKSAAPª, K = 2	58.8%	49.8%	52.8%	0.08
CKSAAPª, K = 3	66.0%	41.2%	49.4%	0.07

^a^*CKSAAP* Composition of k-spaced amino acid pairs

**Fig. 3:**
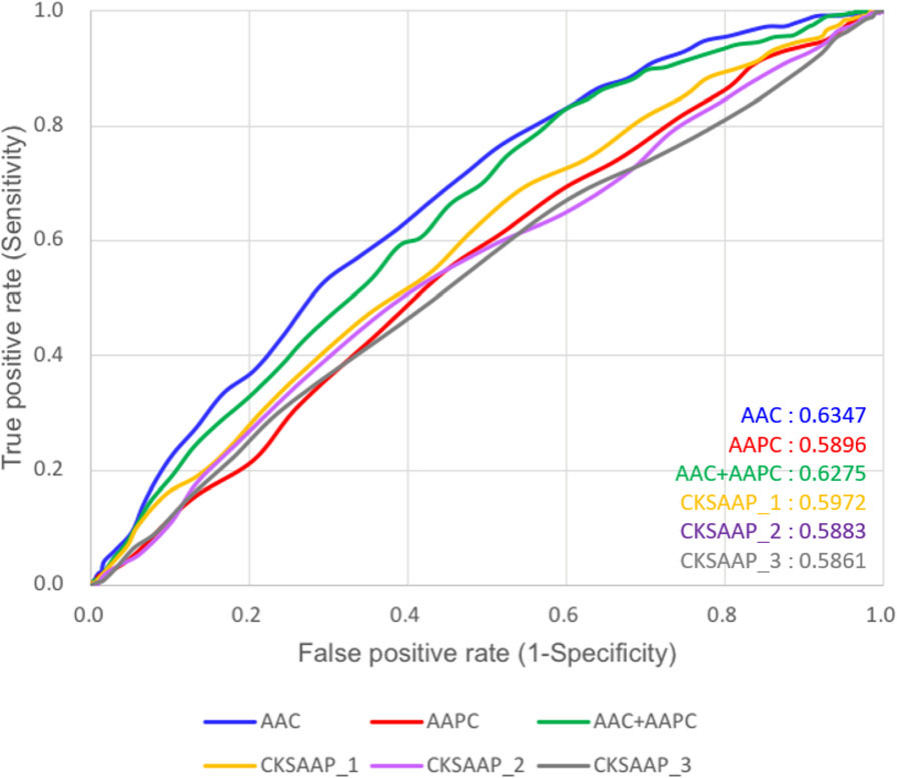
Comparison of ROC curves among the SVM models trained using various features based on five-fold cross-validation

Based on the evaluation of five-fold cross-validation, Fig. 3 presents the comparison of ROC curves between the predictive models trained using various features. The results indicated that the SVM model trained using the AAC feature yielded the best prediction outcomes. To examine the robustness of the predictions from the classification methods, the five-fold cross-validation results of the models were trained by random-forest and decision tree, which also have been provided in Additional files 3 and 4, respectively. According to various evaluation criteria, the SVM model trained using AAC displayed the best overall performance among various predictive models.

### The intrinsic interdependence between positions in substrate sites

**Fig. 4:**
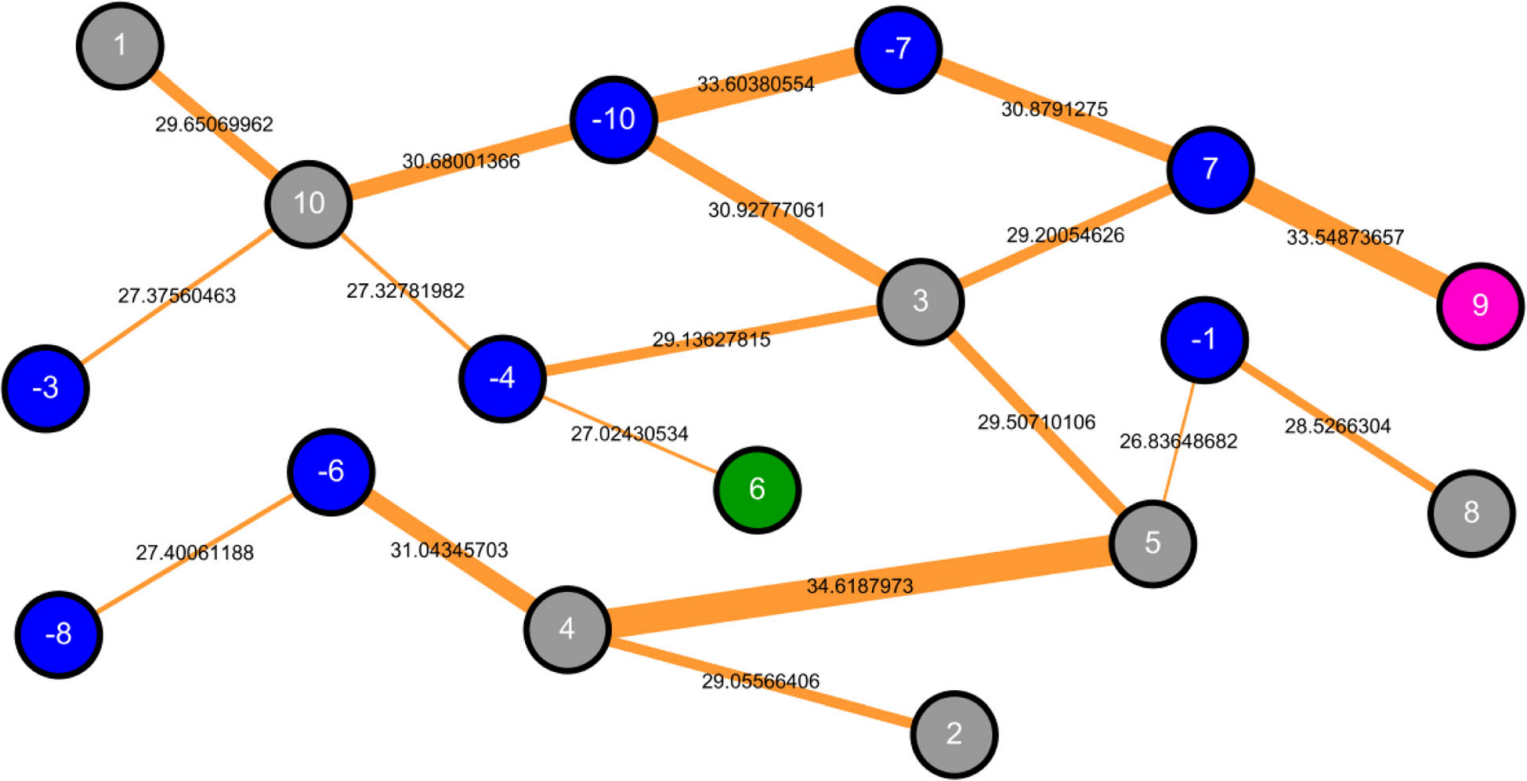
The intrinsic interdependence between positions around glutarylation sites. The number shown in a circle stands for the position around glutarylation site. A higher value displayed on an edge means a more significant dependence of AAC between two circles (positions)

In this study, we used a dependency graph method to fully display the intrinsic interdependence between positions in glutarylation sites. As shown in Fig. 4, the highest chi-square test result was obtained between position + 4 and + 5, indicating a strong interdependence between the two positions. As inferred from Fig. 2c, positively charged amino acids are frequently found in the upstream region of glutarylated sites, at positions starting from − 10 to − 1. A strong interdependence between positions − 10 and − 7 was also observed, according to the upstream consensus region of glutarylation sites. Similarly, we found that acidic amino acids were enriched at position + 9, and that the pair of amino acids in positions + 9 and + 7 showed strong interdependence, according to the downstream consensus region flanking glutarylation sites. This implies that KAT enzymes recognize substrates likely on conserved motifs of amino acids at specific positions, in agreement with previous biological knowledge.

### MDD-identified motif signatures for glutarylated substrate sites

**Fig. 5:**
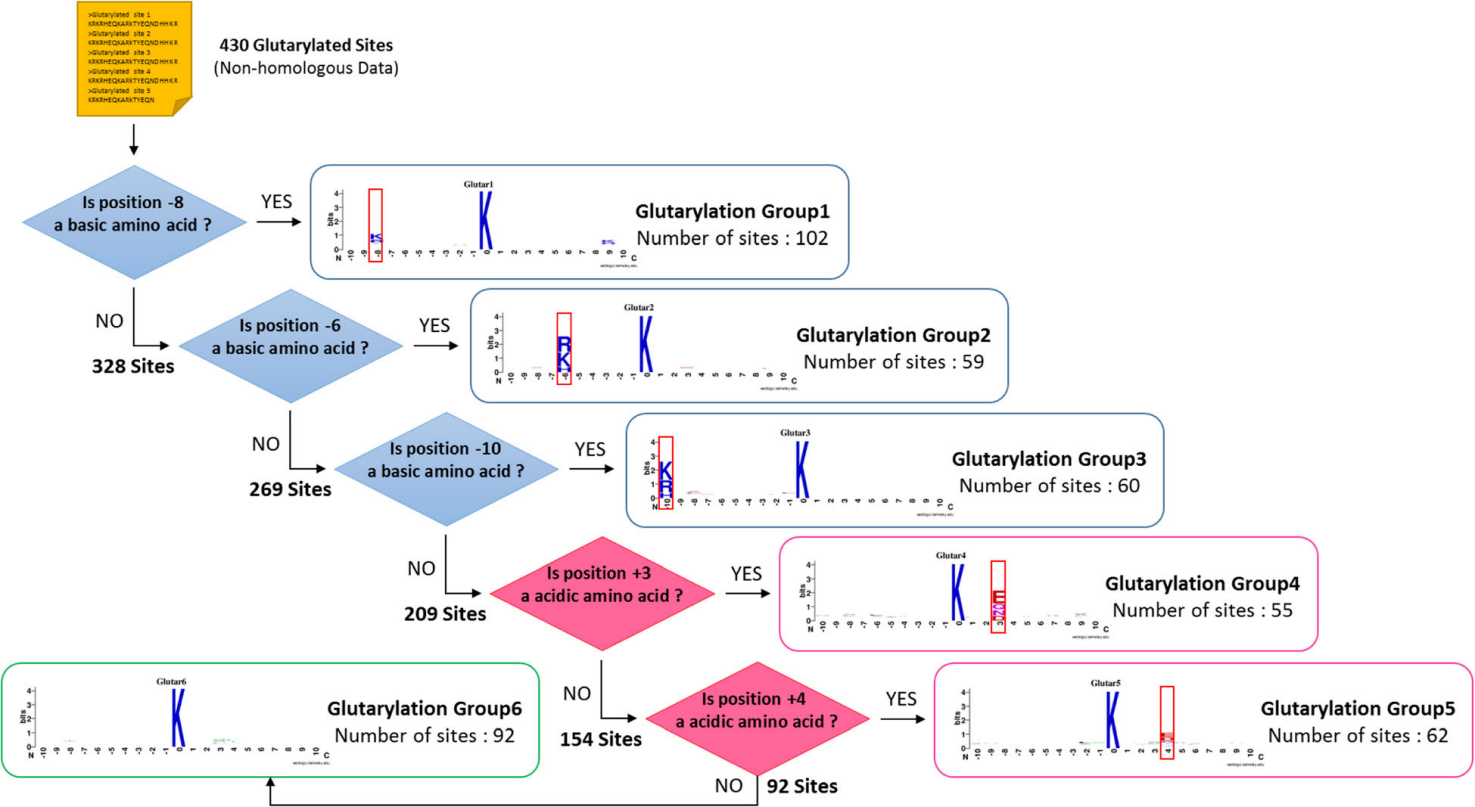
A hierarchical MDD-clustering process on the detection of motif signatures from 430 glutarylated sequences

In this study, MDDLogo was used to explore motif signatures by dividing the positive training dataset (430 sites) into six subgroups. Each subgroup possessed the potential substrate specificity, containing statistically significant dependencies of amino acid composition in specific positions. Fig. 5 provides a tree-like visualization of MDD-clustered subgroups with statistically significant motifs for the 430 non-homologous glutarylation sites. On the left subtree, one motif (subgroup Group1) was detected based on the occurrence of basic amino acids (K, R, and H) at position − 8, with the highest dependence value among all the MDD-clustered subgroups. In parallel, the remaining dataset (328 sites) was further examined for maximal dependency in the occurrence of amino acids at other positions. Subgroup Glutar2 (59 sites) had a similar motif of basic amino acids at position − 6. This result was consistent with the observation in two-sample logo, that basic and hydrophobic amino acids are common in the upstream region of glutarylated lysine. Additionally, subgroups Glutar3 (60 sites) had basic amino acids at position − 10. Subgroups Glutar4 (55 sites) and Glutar5 (62 sites) had acidic amino acids at positions + 3 and + 4, respectively. Finally, the remaining 92 positive sequences formed the sixth subgroup (Glutar6), which contained a slight conservation of amino acids at positions + 3 and + 4.

### Effectiveness of incorporating MDD-identified motifs into the identification of glutarylation sites

**Fig. 6:**
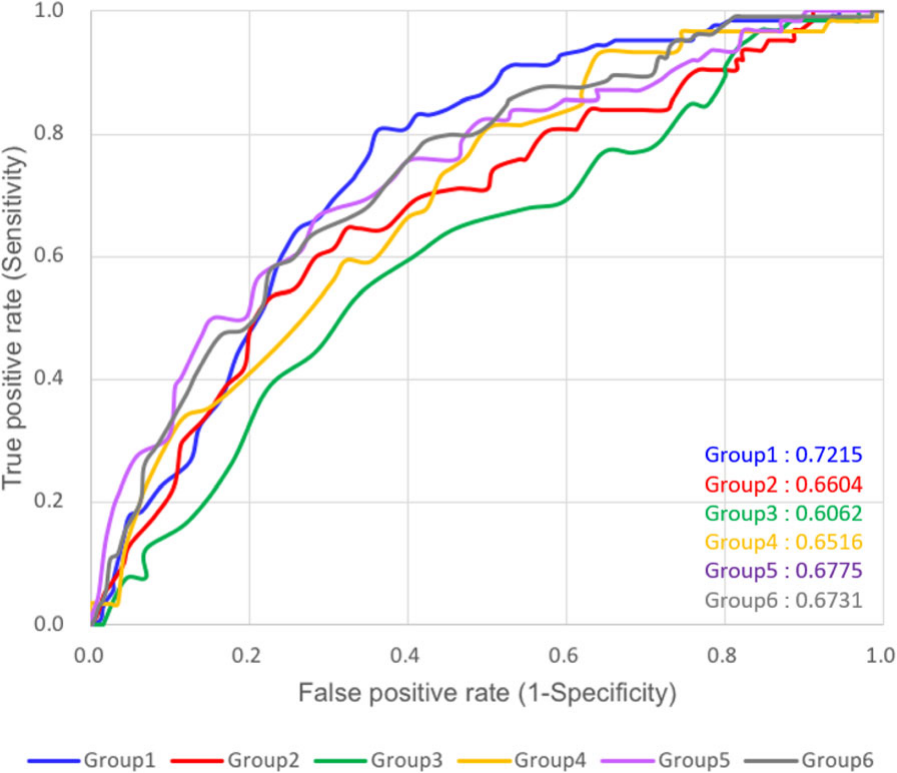
ROC curves of six SVM models trained from MDD-clustered subgroups based on five-fold cross-validation

In order to evaluate the predictive power of MDD-identified substrate motifs in discriminating between glutarylation sites and non-glutarylation sites, LIBSVM was utilized to generate a predictive model for each subgroup based on AAC, the most informative feature. Based on the five-fold cross-validation, Fig. 6 provides the comparison of ROC curves between SVM model trained using all dataset and that trained from MDD-clustered subgroups. In addition, Table 3 provides the predictive sensitivity, specificity, accuracy, and MCC for each subgroup, based on their five-fold cross-validation performance. The values of ROC are also given in Table 3. It shows that subgroup Glutar1 with K/R motif at position − 8 showed a best performance at sensitivity, specificity, accuracy and MCC values of 0.81, 0.64, 0.69, and 0.42, respectively. Subgroup Glutar6 had predictive sensitivity, specificity, accuracy, and MCC values of 0.72, 0.63, 0.66, and 0.33, respectively, which is comparable to those of subgroup Glutar1. Subgroup Glutar3, whose motif was only slightly conserved, performed badly in general with relatively low sensitivity, 0.60; specificity, 0.59; accuracy, 0.60; and MCC, 0.18. Overall, the six subgroups, which contained conserved motifs of amino acids at specific positions, yielded promising accuracy as well as a balanced sensitivity and specificity. To use the information provided by all six motifs to identify glutarylation sites with substrate specificity, the six SVM models trained from MDD-clustered subgroups were incorporated into an integrated SVM model. The values of probability estimated from five SVM models according to a specific motif signature were combined to form an input vector for the integrated SVM classifier. As shown in Table 3, based on five-fold cross-validation, the predictive performance of the integrated SVM model was significantly improved as compared to that of the single SVM model trained from all datasets without MDD clustering. The integrated SVM model presented a sensitivity, specificity, accuracy, and MCC of 0.68, 0.62, 0.64, and 0.28, respectively. In summary, the integrated SVM model combining all MDD-identified motif signatures can enhance the performance of glutarylation site identification and could be implemented as a web-based prediction resource.

**Table 3:** Five-fold cross-validation results for six SVM models trained from MDD-identified motifs

Dataset	Number of positive data	Number of negative data	Sn	Sp	Acc	MCC
All Data	430	860	62.0%	61.3%	61.6%	0.22
Glutar 1	102	204	80.6%	63.7%	69.4%	0.42
Glutar 2	59	120	64.5%	67.7%	66.7%	0.31
Glutar 3	60	120	60.0%	59.2%	59.5%	0.18
Glutar 4	55	110	66.1%	60.2%	62.1%	0.25
Glutar 5	62	121	75.8%	59.7%	65.1%	0.33
Glutar 6	92	185	72.1%	62.5%	65.7%	0.33
Combined result	430	860	67.7%	61.9%	63.8%	0.28

### Implementation of MDDGlutar web interface

**Fig. 7:**
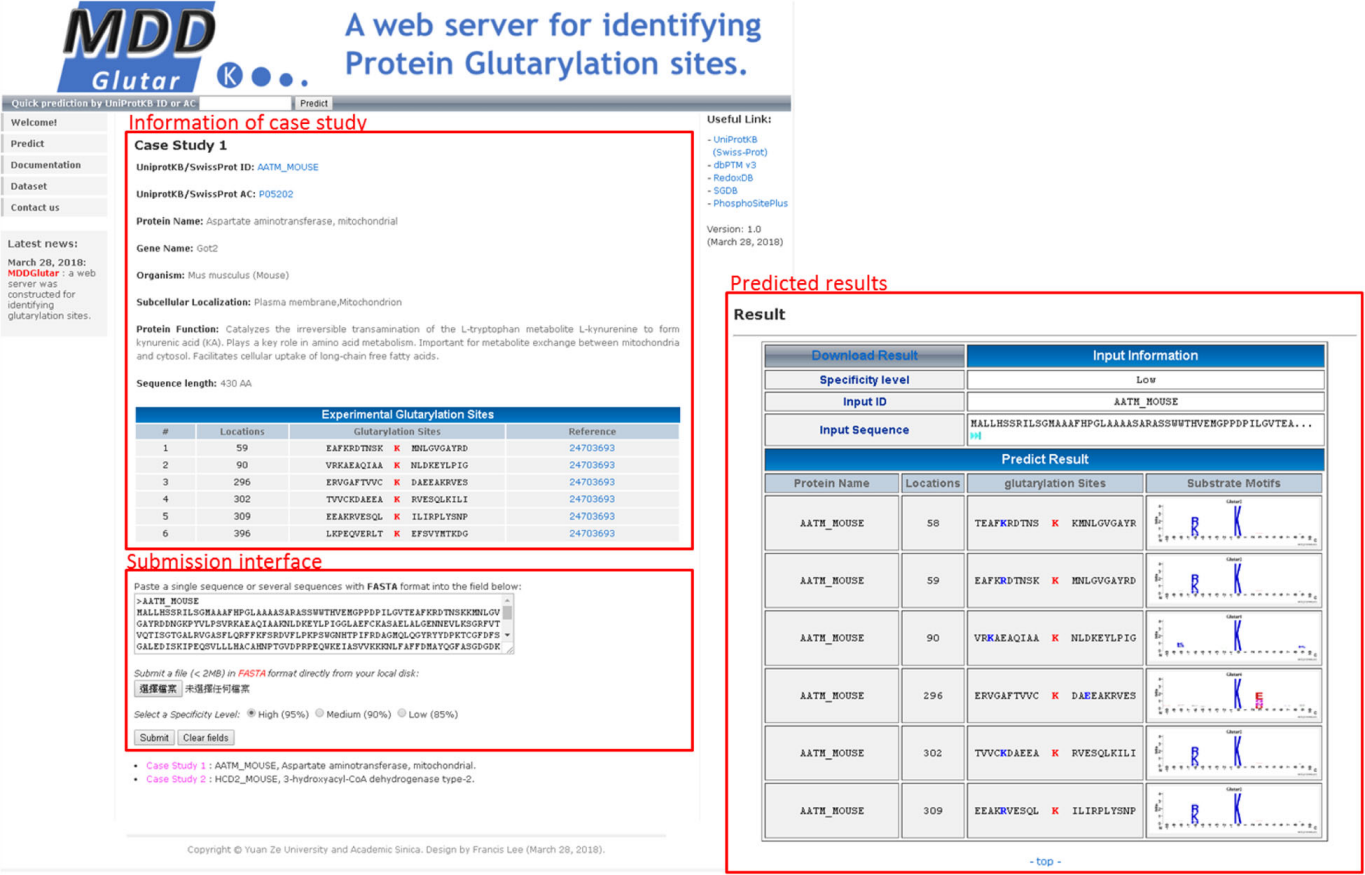
A case study of glutarylation site prediction on mouse aspartate aminotransferase (UniProt ID: AATM_MOUSE)

Because experimentation is a time-consuming and labor-intensive process, development of an effective prediction system can aid the study of glutarylation sites. However, no method dedicated to the characterization of potential substrate motifs of glutarylated sites currently exists. Thus, we were inspired to develop a user-friendly web tool, named MDDGlutar, for the identification of glutarylation sites with their substrate motifs. The generated SVM model, combining all MDD-identified substrate motifs and the AAC feature set, was adopted to implement the prediction function on the website. After submitting protein sequences in the FASTA format, MDDGlutar returns the prediction results, including glutarylation sites, their flanking amino acids, and the corresponding substrate motif signatures. A case study on mouse aspartate aminotransferase (UniProt ID:AATM_MOUSE) was utilized to demonstrate the effectiveness of MDDGlutar. The mouse aspartate aminotransferase contains six verified glutarylation sites at Lys-59, Lys-90, Lys-296, Lys-302, Lys-309, and Lys-396 [9]. As presented in Fig. 7, MDDGlutar could achieve an accurate prediction at the six validated glutarylation sites, according to the corresponding motif signatures.

## Discussions

An independent test set of glutarylation sites was also taken from PLMD [16], which consisted of 46 positive sites and 92 negative sites. It was used to further evaluate the predictive power of the single SVM model trained using all the training data and the integrated SVM model trained using the six MDD-identified motifs. As shown in Table 4, the single SVM model yielded a sensitivity of 0.609, a specificity of 0.685, an accuracy of 0.659, and an MCC of 0.28. Meanwhile, the performance of the integrated SVM model achieved a sensitivity of 0.652, a specificity of 0.739, an accuracy of 0.710, and an MCC of 0.38. However, the prediction ability of the proposed method, upon independent testing, showed that it can outperform other prediction methods at a specified level of false positive rate (1-specificity).

**Table 4:** Performance comparison between proposed methods and an existing tool (GlutPred) based on independent testing dataset

Methods	TP	FN	TN	FP	Sn	Sp	Acc	MCC
Single SVM	28	18	63	29	60.9%	68.5%	65.9%	0.28
Integrated SVM	30	16	68	24	65.2%	73.9%	71.0%	0.38
GlutPred	25	21	84	8	54.3%	91.3%	79.0%	0.50

To further demonstrate the effectiveness of the proposed model, the independent test dataset was used to compare the model with existing prediction tool. Considering previously published prediction tools, only one predictor of glutarylation sites, GlutPred [55], was freely available. We compared our predictive performance to that of GlutPred based on independent testing datasets. As it can be seen in Table 4, GlutPred yielded higher specificity than our model, i.e., 0.91. However, the high true-negative prediction of GlutPred resulted in lower sensitivity in the identification of glutarylation sites. It is worth noting that the comparison cloud was controversial because the same data were used as source for the two studies, which means that a majority of the testing data was also present in the training data of GlutPred. However, although the proposed method could not provide better specificity than GlutPred, the results of the independent testing demonstrated that the integrated SVM model (MDDGlutar) could provide a promising performance with balanced sensitivity and specificity in the prediction of glutarylation sites.

## Conclusion

In this study, we proposed a bioinformatics method for characterization and identification of glutarylation sites using substrate site specificity. The investigation using two-sample logo revealed that the most conspicuous feature of glutarylation sites is an enrichment of positively charged amino acids (K and R) upstream of the glutarylated lysine, as well as of basic amino acids at positions − 10. Based on five-fold cross-validation, the SVM model trained with the feature AAC achieved the highest sensitivity, specificity, accuracy, and MCC. As stated previously, the main purpose of this study was to explore the substrate motifs of glutarylation sites based on amino acid sequences. First, we measured the interdependence between two positions to fully capture the intrinsic interdependence in the neighboring region of glutarylation sites. After application of MDDLogo on positive training dataset, the glutarylated sequences were clustered into six subgroups corresponding to statistically significant motif signatures. The MDD-identified motifs could thus be employed to develop an integrated SVM model, significantly enhancing the predictive performance of glutarylation sites identification. An independent testing dataset was further prepared for evaluating two models: the integrated SVM model and the single SVM model without MDD implementation. The independent testing results demonstrated that the integrated model, which combined six MDD-identified motifs, provided a better predictive performance with balanced sensitivity and specificity. Consequently, the proposed model was employed to build a web-based resource, named MDDGlutar, to identify glutarylation sites and their corresponding substrate motifs.

## Additional files

Additional file 1: **Table S1.** Performance comparison among the SVM models trained using different window lengths. (DOCX 13 kb)

Additional file 2: **Table S2.** Results of k-fold cross-validation with *k* ranging from 5 to 10. (DOCX 15 kb)

Additional file 3: **Table S3.** Five-fold cross validation results of Random Forest models trained using various features. (DOCX 14 kb)

Additional file 4: **Table S4.** Five-fold cross validation results of Decision Tree classifiers trained using various features. (DOCX 14 kb)

## Abbreviations

AAC: Amino acid composition
AAPC: Amino acid pair composition
Acc: Accuracy
CKSAAP: Composition of k-spaced amino acid pair
KAT: Lysine acyltransferase
KDACs: Lysine deacylases
MCC: Matthews correlation coefficient
MDD: Maximal dependence decomposition
PLMD: Protein lysine modifications database
PTM: Post-translational modification
RBF: Radial basis function
Sn: Sensitivity
Sp: Specificity
SVM: Support vector machine
