# Supplementary material for: Characterization and identification of lysine glutarylation based on intrinsic interdependence between positions in the substrate sites
Source: BMC Bioinformatics. 2019 Feb 4;19(Suppl 13):384. doi: 10.1186/s12859-018-2394-9 (PMC7394328; doi:10.1186/s12859-018-2394-9)
Supplement: Supplementary file 1 — Table S1. Performance comparison among the SVM models trained using different window lengths. (DOCX 13 kb) [file 12859_2018_2394_MOESM1_ESM.docx]

Table S1 Performance comparison among the SVM models trained using different window lengths.

| **Window length** | **TP** | **FN** | **TN** | **FP** | **Sn** | **Sp** | **Acc** | **MCC** |
| --- | --- | --- | --- | --- | --- | --- | --- | --- |
| 11 | 223 | 207 | 512 | 348 | 51.9% | 59.5% | 57.0% | 0.11 |
| 13 | 259 | 171 | 437 | 423 | 60.2% | 50.8% | 54.0% | 0.10 |
| 15 | 266 | 164 | 510 | 350 | 61.9% | 59.3% | 60.2% | 0.20 |
| 17 | 267 | 163 | 513 | 347 | 62.1% | 59.7% | 60.5% | 0.21 |
| 19 | 265 | 165 | 504 | 356 | 61.6% | 58.6% | 59.6% | 0.19 |
| 21 | 265 | 165 | 535 | 325 | 61.6% | 62.2% | 62.0% | 0.23 |
| 23 | 262 | 168 | 535 | 325 | 61.0% | 62.2% | 61.8% | 0.22 |
| 25 | 242 | 188 | 610 | 350 | 56.3% | 59.3% | 58.3% | 0.15 |
