# Supplementary material for: Characterization and identification of lysine glutarylation based on intrinsic interdependence between positions in the substrate sites
Source: BMC Bioinformatics. 2019 Feb 4;19(Suppl 13):384. doi: 10.1186/s12859-018-2394-9 (PMC7394328; doi:10.1186/s12859-018-2394-9)
Supplement: Supplementary file 2 — Table S2. Results of k-fold cross-validation with k ranging from 5 to 10. (DOCX 15 kb) [file 12859_2018_2394_MOESM2_ESM.docx]

Table S2 Results of *k*-fold cross-validation (CV) with *k* ranging from 5 to 10.

| ***k*-fold CV** | **TP** | **FN** | **TN** | **FP** | **Sn** | **Sp** | **Acc** | **MCC** |
| --- | --- | --- | --- | --- | --- | --- | --- | --- |
| 5-fold | 265 | 165 | 535 | 325 | 61.6% | 62.2% | 62.0% | 0.23 |
| 6-fold | 263 | 167 | 513 | 347 | 61.2% | 59.7% | 60.2% | 0.20 |
| 7-fold | 254 | 176 | 527 | 333 | 59.1% | 61.3% | 60.5% | 0.19 |
| 8-fold | 260 | 170 | 540 | 320 | 60.5% | 62.7% | 62.0% | 0.22 |
| 9-fold | 278 | 152 | 518 | 342 | 64.7% | 60.2% | 61.7% | 0.23 |
| 10-fold | 262 | 168 | 532 | 328 | 60.9% | 61.95 | 61.6% | 0.22 |
