# Supplementary material for: Characterization and identification of lysine glutarylation based on intrinsic interdependence between positions in the substrate sites
Source: BMC Bioinformatics. 2019 Feb 4;19(Suppl 13):384. doi: 10.1186/s12859-018-2394-9 (PMC7394328; doi:10.1186/s12859-018-2394-9)
Supplement: Supplementary file 3 — Table S3. Five-fold cross validation results of Random Forest models trained using various features. (DOCX 14 kb) [file 12859_2018_2394_MOESM3_ESM.docx]

Table S3 Five-fold cross validation results of Random Forest models trained using various features.

| **Training features** | **Sensitivity** | **Specificity** | **Accuracy** | **MCC** |
| --- | --- | --- | --- | --- |
| Amino Acid Composition (AAC) | 59.9% | 56.5% | 57.6% | 0.15 |
| Amino Acid Pair Composition (AAPC) | 46.6% | 44.9% | 45.4% | -0.08 |
| CKSAAP , K=1 | 53.4% | 49.4% | 50.7% | 0.03 |
| CKSAAP , K=2 | 52.1% | 42.0% | 45.4% | -0.06 |
| CKSAAP , K=3 | 51.5% | 42.6% | 45.6% | -0.06 |

*CKSAAP, Composition of k-spaced amino acid pairs.
