# Supplementary material for: Characterization and identification of lysine glutarylation based on intrinsic interdependence between positions in the substrate sites
Source: BMC Bioinformatics. 2019 Feb 4;19(Suppl 13):384. doi: 10.1186/s12859-018-2394-9 (PMC7394328; doi:10.1186/s12859-018-2394-9)
Supplement: Supplementary file 4 — Table S4. Five-fold cross validation results of Decision Tree classifiers trained using various features. (DOCX 14 kb) [file 12859_2018_2394_MOESM4_ESM.docx]

Table S4 Five-fold cross validation results of Decision Tree classifiers trained using various features.

| **Training features** | **Sensitivity** | **Specificity** | **Accuracy** | **MCC** |
| --- | --- | --- | --- | --- |
| Amino Acid Composition (AAC) | 55.3% | 54.2% | 54.6% | 0.09 |
| Amino Acid Pair Composition (AAPC) | 49.2% | 47.4% | 48.0% | -0.03 |
| CKSAAP , K=1 | 50.4% | 47.5% | 48.5% | -0.02 |
| CKSAAP , K=2 | 49.2% | 48.3% | 48.6% | -0.02 |
| CKSAAP , K=3 | 48.5% | 47.7% | 48.0% | -0.04 |

*CKSAAP, Composition of k-spaced amino acid pairs.
